# Spatially varying associations between the built environment and older adults' propensity to walk

**DOI:** 10.3389/fpubh.2022.1003791

**Published:** 2022-08-26

**Authors:** Chunmei Yang, Xianglong Tang, Linchuan Yang

**Affiliations:** ^1^School of Physical Education, Southwest Jiaotong University, Chengdu, China; ^2^Department of Urban and Rural Planning, School of Architecture, Southwest Jiaotong University, Chengdu, China

**Keywords:** population aging, physical environment, street greenery, walking behavior, travel behavior, geographically weighted regression, spatial heterogeneity, spatial non-stationarity

## Abstract

Population aging has become a severe issue facing most nations and areas worldwide—with Hong Kong being no exception. For older adults, walking is among the most well-liked travel modes, boosting their overall health and wellbeing. Some studies have confirmed that the built environment has a significant (spatially fixed) influence on older adults' walking behavior. However, little consideration has been given to the potential spatial heterogeneity in such influences. Hence, this study extracted data on older adults' (outdoor) walking behavior from the 2011 Hong Kong Travel Characteristics Survey and measured a series of built environment attributes based on geo-data (e.g., Google Street View imagery). Logistic regression and geographically weighted logistic regression models were developed to unveil the complicated (including spatially fixed and heterogeneous) association between the built environment and older adults' propensity to walk. We show that population density, land-use mix, street greenery, and access to bus stops are positively connected with the propensity to walk of older adults. Intersection density seems to impact walking propensity insignificantly. All built environment attributes have spatially heterogeneous effects on older adults' walking behavior. The percentage of deviance explained is heterogeneously distributed across space.

## Introduction

Population aging has become a pressing global concern (1). According to the United Nations, by 2030, 2050, and 2100, the number of the world's older populations (aged 65 years or above) will reach 1, 1.30, and 2.46 billion, respectively. Along with the sharp increase in absolute numbers, the ratio of older adults to the total population is constantly booming. This figure reached 9.1% in 2019 and is projected to increase to 11.7, 15.9, and 22.6% in 2030, 2050, and 2100, respectively (2). Hong Kong, a global city with ~7.5 million inhabitants, shares this issue with other metropolises worldwide. In 2020, the proportion of the older population aged 65 years or above was 18.2%, second in Asia and only behind Japan (28.4%, the highest in the world). Additionally, it is anticipated that the proportion of the older population in Hong Kong will progressively increase and is predicted to rise to 34% in 2049.

It is commonly acknowledged that older adults' quality of life is highly correlated with their mobility, a lack of which leads to lower overall health and wellbeing. Daily mobility is a prerequisite for improving personal life, promoting social engagement, and enhancing emotional health (3). Particularly, older adults become less socially engaged because of life cycle stage changes (e.g., retirement) or aging-related events (e.g., death of the spouse) (4). Conceivably, people need to travel (mobility) to access urban services and engage in social activities, ultimately affecting their quality of life.

Walking has been widely recommended due to its economic, environmental, social, safety, and health benefits (5, 6). Walking helps reduce the prevalence of cardiac disease, psychosis, Alzheimer's disease, and hypertension (7–10). Furthermore, walking encourages social engagement, activity involvement, and interpersonal communication, contributing to active aging (11). Therefore, encouraging walking activities among older adults is essential for enhancing their quality of life. Furthermore, in many Chinese cities, limited car access makes walking a critical and indispensable travel mode for older adults (11, 12). This is especially true for Hong Kong, a city famous for its diverse land uses, pedestrian-friendly urban planning, and high walkability (13).

The built environment is a significant component of the geographic environment and has received enormous scholarly attention from many disciplines, including urban planning, geography, transportation, public health, psychology, and GIS (14–20). The most popular of these assessment methods is the “3Ds”/“5Ds”/“7Ds” model, which categorizes the built environment attributes into either three, five, or seven dimensions (21, 22). Numerous studies have revealed that the built environment profoundly affects individuals' travel behavior (23), especially for older adults who prefer to travel short distances due to their functional and cognitive limitations (24). They have demonstrated that a walkable and mixed urban form, green spaces, and parks are fundamental to their walking behavior (25–27).

Identifying the complex relationships between the built environment and older adults' walking behavior is the primary step toward spatial intervention. However, existing studies have emphasized the spatially fixed correlation between built environment attributes (e.g., street greenery and land-use mix) and older adults' walking behavior while largely ignoring the potential presence of spatial heterogeneity. Exploring whether there is a spatially heterogeneous connection between the built environment and older adults' propensity to walk is a crucial point of discussion. A fuller understanding of this connection serves as an indispensable and crucial reference for targeted treatments implemented to encourage walking activity among older adults. Following previous studies on the spatial heterogeneity in the connection between travel behavior and the built environment (28, 29), this study hypothesizes that there are spatially heterogeneous associations between older adults' propensity to walk and built environmental variables. It extracted the socio-demographic and walking behavior data from the 2011 Hong Kong Travel Characteristics Survey (HKTCS) and measured five built environment attributes based on multi-source geo-data. Notably, Google Street View (GSV) imagery was assessed through fully convolutional neural networks to quantify street greenery. Besides, we developed a logistic regression model to analyze the global relationship and establish a geographically weighted logistic regression (GWLR) model [a member of the geographically weighted regression (GWR) family] to scrutinize the local relationship and the presence of spatial heterogeneity in the relationship.

This study contributes to the literature by (1) exploring the global association between built environment characteristics and older adults' propensity to walk, (2) spurring further understanding of the spatial heterogeneity in this connection, and (3) serving as a reference for studies exploring whether there is spatial heterogeneity between the built environment and people's travel patterns.

The remainder of this paper proceeds as follows. Section “Literature review” reviews related studies on the built environment and older adults' mobility/travel behavior. Section “Data” presents the HKTCS 2011 data and built environment data. Section “Methodology” introduces the logistic regression model, the GWLR model, and the variables used in this study. Section “Results” reveals the global and local modeling results. Section “Conclusions and discussion” concludes the paper and discusses the implications.

## Literature review

Existing studies mainly use the travel survey data collected by the government or researchers and apply econometric models (e.g., linear regression models, discrete choice models, and structural equation models) to identify the factors influencing people's travel outcomes and to unveil and assess the marginal effects or elasticities of attributes which play a decisive role. Travel outcomes consist of travel frequency, travel propensity (whether to travel or not), travel time or distance, walking duration, walking frequency, the propensity to walk (whether to walk or not), transit travel frequency, etc.

The factors that either promote or hinder people's travel behavior can be roughly categorized into individual or household socio-demographic characteristics and the built environment. Other factors such as the social environment, transit service attributes (e.g., priority seats and free bus passes), attitudes, and preferences received little scholarly attention.

### Individual or household socio-demographic characteristics

Previous studies often adopt several variables to comprehensively capture, characterize, and control the socio-economic attributes of individuals or households, in which both age and gender are the factors receiving the most attention. Some studies have suggested the consistent effect of age on older adults' mobility and travel behavior. That is, with advancing age, the mobility of older adults generally decreases because of the degradation of physical function. For example, the older the population in Hong Kong is, the more reluctant they are to go out (30) and the lower trip frequency (31), which coincides with the evidence gathered from studies in Washington (32), Hamilton (33), and London (34).

The effect of gender on the mobility of older adults remains controversial. Yang et al. (30) concluded that male seniors in Hong Kong are more likely to go out than their female counterparts. By contrast, Kim and Ulfarsson (35) concluded that the travel frequency by motor, bus, and para-transit of female seniors in Washington is higher than that of their male counterparts.

Conflicting evidence on the correlation between older adults' educational attainment and mobility exists. Evans (36) and Kim (32) deemed that older adults who have a higher education level are more inclined to travel. However, Feng's (37) research elaborated that in Nanjing, there is no significant discrepancy between the activity frequency of older adults with elementary school education and those with college education or higher.

The effect of car availability on the mobility of older adults has also received substantial scholarly attention. Yang and Cui (31) hold that motor ownership displayed no association with older adults' mobility in Hong Kong, coinciding with the Nanjing-based findings of Feng et al. (38, 39) and Feng (37). However, this outcome differs from the findings presented in many studies, especially those from North America, Oceania, and Europe, where scholars such as Schwanen et al. (40), Paez et al. (33), and Roorda et al. (41) emphasized that having a car facilitates older adults' mobility. The reason for such discrepancy is understandable. In car-dominant or car-centric cities, most people regard cars as a decisive means of mobility, which is largely different from transit-dependent cities in East Asia such as Beijing, Hong Kong, and Singapore, where the high transit market share resulted in a low significance of car ownership on older adults' mobility.

Other socio-demographic factors such as household size (39), monthly household income (42), employment status (30), job type (39), mobile phone ownership (43), living alone or not (31), housing property rights (36), and race (36) have also received some scholarly attention.

### Built environment attributes

Recently, the impact of the built environment on older adults' travel behavior and mobility has increasingly attracted much attention. Population density, leisure facility density, transit accessibility, and green space are oft-discussed built environment attributes. Analysis methods consist of linear regression, discrete choice models, order logit/probit regression, Poisson or negative binomial regression, structural equation models, and the models incorporating spatial autocorrelation, spatial heterogeneity, and variable hierarchy (e.g., spatial regression models and the GWR model). Moreover, these built environment attributes are mainly measured through GIS analysis and field survey, with a small portion of the studies using street view imagery.

Most of these studies looked at North America (the USA and Canada). Evans (36) utilized stepwise discriminant analysis to identify characteristics impacting the travel propensity of older adults without a vehicle and older adults aged 75 years or above using data extracted from the 1995 National Personal Transportation Survey. Neighborhood housing density and community environment significantly affected the travel propensity of older adults, while no evidence of associations with travel propensity of older adults was found for variables such as population density and transit accessibility. Kim (32) used structural equation modeling to examine the variables influencing older adults' mobility and concluded that population density and employment density did not have significant effects on mobility (after controlling for individual or household socio-demographic characteristics). Paez et al. (33) found that the location of residence was significantly related to the frequency of non-work trips among older adults in Hamilton, Canada, and that mobility was higher among those living in the eastern and northern regions of the city. Mercado and Páez's modeling results show that population density has insignificant effects on travel distance for older adults in Hamilton, Canada (44). Roorda et al. analyzed the mobility of those who were transportation disadvantaged in three Canadian cities using the ordered probit model. They found that population density improves the mobility of the transportation disadvantaged (including older adults) in Montreal but gets otherwise inhibited (41).

Scholars have also conducted similar research in both the European and Australian contexts. Based on the outcomes of the order probit model, Schmöcker et al. concluded that older adults living in central London travel less frequently than those living in the suburbs. However, when conducting personal business, few differences are seen in the travel frequency between the two groups (34). Su and Bell used a nested logit model to scrutinize the variables affecting the travel mode choice of older adults in London and found that bus stop density and service frequency play a decisive role (45). In the Australian city of Adelaide, Truong and Somenahalli examined the variables determining the frequency of transit usage by older adults and concluded that access to the city center and station density had a negligible impact (43). Additionally, Pettersson and Schmöcker discovered that in Manila, Philippines, older adults made more journeys as population density rose (46).

Some studies have recently used Chinese cities as study areas as well. According to Feng et al., population density had no discernible effect on the travel frequency among older adults in Nanjing, but subway accessibility did (39). Yang's analysis of HKTCS data revealed that older adults' trip frequency is significantly impacted by transit accessibility (31). Unlike intersection density (which had a negative impact) and land use entropy (which had an insignificant impact), population density and retail store density had a positive influence on older adults' propensity to walk in Hong Kong; population density significantly enhances older adults' walking duration, while street greenery positively affected both the propensity to walk and walking duration (47). Using the Poisson and negative binomial models, Yang and Cui discovered that transit accessibility positively affects the travel frequency of older adults in Hong Kong, whereas road network density plays a negative role. Furthermore, they identified that transit accessibility has a considerable impact on the travel frequency of younger seniors (60–75 years) but not on that of older ones (75 years or above) (31). Yang et al. clarified a significant effect of street greenery on older adults' propensity to travel in Hong Kong using data from GSV imagery (30). However, Cheng et al. indicated that residential location in Nanjing had a greater effect on older adults' travel behavior than it did on younger adults (48).

Some studies concentrated on the built environment with Chinese characteristics and reached conclusions distinct from Western studies, thereby enhancing the West-dominated senior mobility research. Feng et al. assessed the travel distance and frequency of older adults in Nanjing. They discovered that the residents of unitary welfare housing traveled frequently but were still far less than those of mixed communities and regular commercial housing estates (38). Similarly, Feng adopted ordered logit and linear regression models to scrutinize the correlation of the built environment with older adults' travel based on the 2012 Nanjing Resident Travel Survey data and concluded that chess rooms and parks/plazas are more attractive to older adults than facilities such as gymnasiums and museums (37). However, Cheng et al. identified what factors are related to walking/cycling frequency and duration of older adults in Nanjing and determined that park/plaza and chess room accessibility have a significant effect, unlike market and gym accessibility which did not show any correlation (11).

### Thrust of this study

To our knowledge, senior travel behavior and mobility research first appeared in developed countries (e.g., the US and Canada). A compelling explanation is that population aging happens very early in such countries, facilitating social concerns about related issues. However, the social characteristics of Western countries starkly differ from those of China. For instance, many Western countries (e.g., the US, Canada, and Australia) feature low-density urban development, single land use, the predominance of car-based transport, and a meager transit share. Chinese cities are generally featured by high density, mixed land use, well-developed transit systems, and high transit share. Therefore, the Western experience cannot be directly transposed in China because of vastly different social and urban traits.

Related research has been recently conducted in China. Much scholarly attention has been given to variables that affect older adults' travel behavior. Generally, their results are relatively dispersed, and there remains a lack of locally focused research to seek out more insightful solutions. Besides, the spatial heterogeneity in the connection between the built environment and older adults' travel behavior has received insufficient scholarly attention, which is the primary focus and a key task of this study.

## Data

### Travel data

The Transport Department of Hong Kong conducts the HKTCS, a thorough and detailed periodical travel survey. The latest was carried out from September 2011 to January 2012. It consisted of three main dimensions: the Household Interview Survey to gather 24-h travel data from Hong Kong residents, the Stated Preference Survey to determine the variables influencing people's choice of transportation mode, and the Hotel/Guesthouse Tourists Survey to collect travel data from guests staying in hotels/guesthouses.

The Household Interview Survey, similar to many comprehensive government travel surveys, had three dimensions: (1) household data (e.g., residence location, household size, and residence type); (2) household member data (e.g., gender); and (3) 24-h travel data. Additionally, HKTCS 2011 included the respondents' residence location information, allowing the geocoding of the data in the ArcGIS Pro (version 2.8) platform for residence-centered built environment assessments.

**Figure 1:**
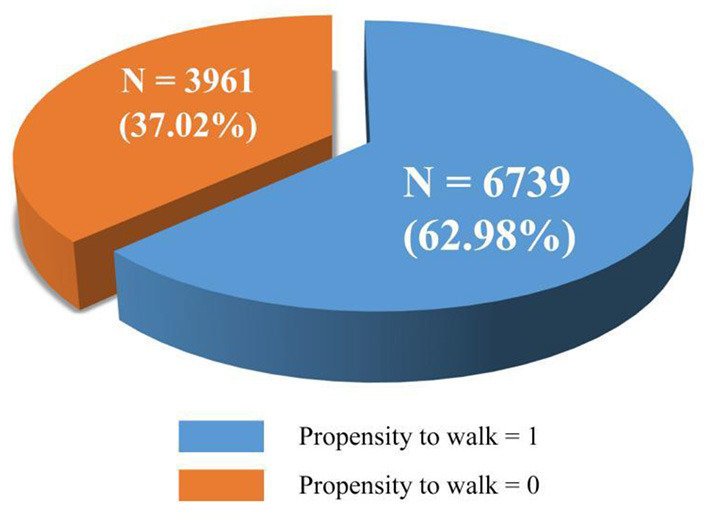
Propensity to walking details of the sampled older adults.

**Figure 2:**
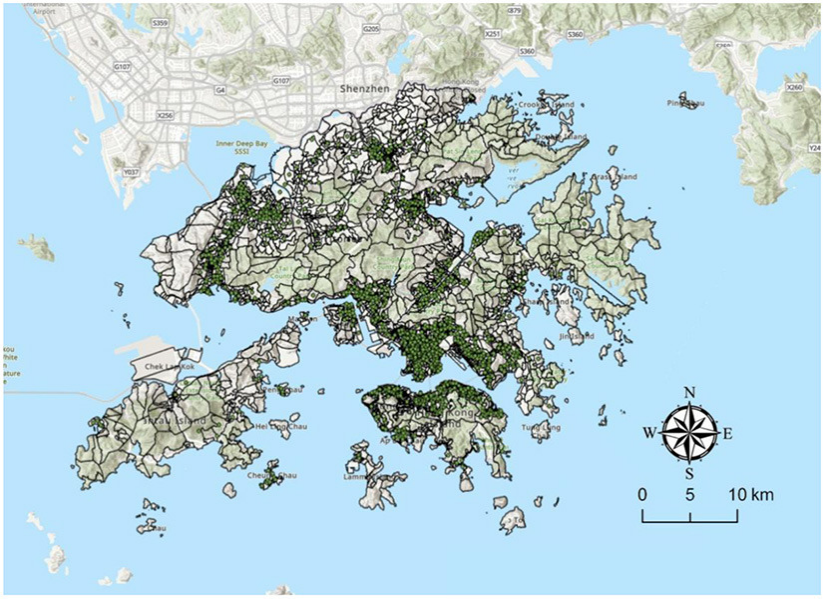
The spatial distribution of the sampled older adults.

Following international standards, this study extracted walking behavior data and divided the older adults (65 years or above) into two groups based on their propensity to walk: those who made at least one walking trip within 24 h (propensity to walk = 1) and those who did not (propensity to walk = 0; Figure 1). Figure 2 reveals the spatial distribution of the sampled older adults (*N* = 10,700).

### Street greenery data and other built environment data

Given its perspective that is highly comparable to human vision, street-view imagery precisely captures a 360° high-resolution panoramic view of the physical urban environment. Compared to conventional data sources, it has the unique strengths of high geographic coverage, low data bias, cost-effectiveness, and human-centeredness (49). Introduced in 2007, GSV was one of the first online street-view services and has since covered cities in roughly 90 countries (50). GSV data are mainly collected by GPS-equipped sensing vehicles.

**Figure 3:**
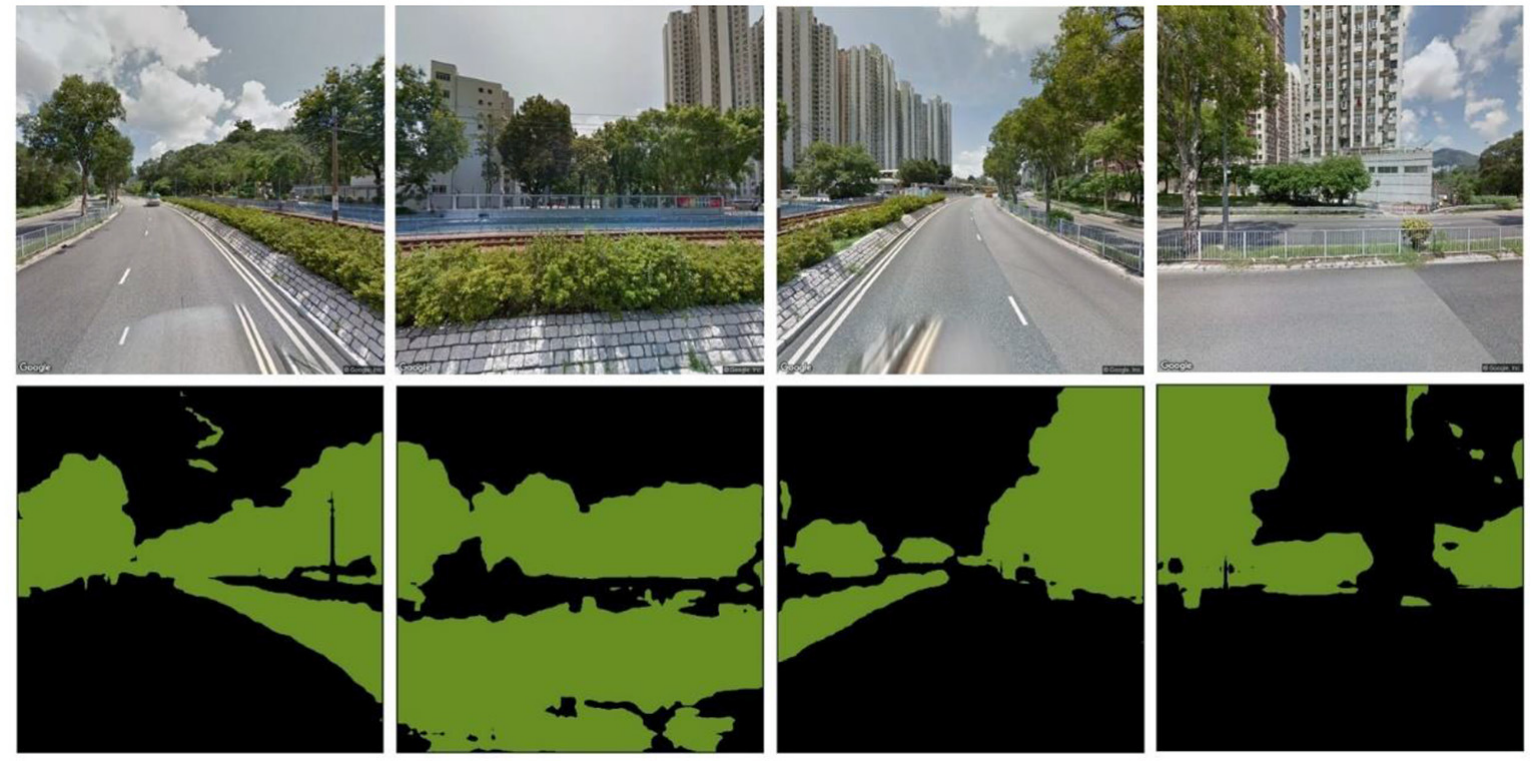
The estimation of the green view index.

Eye-level street greenery, which reflects actual pedestrian perceptions of street greenery, has been proven more relevant to people's active travel than other green space measures (30). Hence, this study assessed the eye-level green view index using GSV imagery to stimulate people's perception of street greenness as follows: first, the residence locations were geocoded into the ArcGIS platform. Second, all street segments in the vicinity of the geocoded locations were automatically identified. Third, the generated locations of GSVs were automatically created with a fixed spacing of 50 m and then recorded in the coordinates. Fourth, the matching GSV imagery was downloaded. For each point, four images together represented a 360° panorama (51). Last, fully convolutional neural networks (FCN-8s) were applied to investigate greenfield pixels (Figure 3) (52). The formula for the green view index at a GSV generation location is,


(1) $$\begin{array}{l} Green\;view\;index\;=\;\frac{\sum_{i=1}^{4}Greenery\;pixels_{i}}{\sum_{i=1}^{4}Total\;pixels_{i}} \end{array}$$

The built environment was assessed in the ArcGIS Pro platform based on the “3Ds” built environment evaluation framework and available data. POIs (points of interest) and region boundaries were crawled from *OpenStreetMaps*, while land use, TPU (tertiary planning unit)-level data were obtained from the government website.

## Methodology

A logistic regression model was first employed because the propensity to walk of older adults is a binary (dummy, dichotomous, or indicator) variable. Moreover, the availability of location attributes is a prerequisite for adopting the GWLR model to analyze spatial heterogeneity. As an advanced version of the logistic regression model, the GWLR model, using the geographic coordinates of all observations embedded in the data, estimated the spatially varying correlation between the predicted and predictor variables (predictors). Hence, logistic regression models and GWLR models were developed in this study.

### Global model: logistic regression model

Logistic regression analysis is developed to investigate how predictors affect the predicted variable. The logistic regression model is widely developed to examine situations where the predicted variable has exclusively two outcomes. In this study, it was used to link the propensity to walk with the predictors. The model is presented in the form below:


(2) $$\begin{array}{l} P_{i}\;=\;\frac{e^{U_{i}}}{1+e^{U_{i}}} \end{array}$$

Or likewise,


(3) $$\begin{array}{l} U_{i}=logit\left(P_{i}\right)\;=\;\ln\left(\frac{P_{i}}{1-P_{i}}\right) \end{array}$$

where *P*_*i*_ is the likelihood that person *i* takes at least one walking trip, $\frac{P_{i}}{1-P_{i}}$ is often referred to as the odds ratio, and *U*_*i*_ represents the utility of older person *i*, which reflects the determinants affecting person *i*'s trip. The following statement describes the connection between *U*_*i*_ and the predictors:


(4) $$\begin{array}{l} U_{i}\;=\;\beta_{0}\;+\;\sum_{k}\beta_{k}X_{ik}\;+\;\varepsilon_{i} \end{array}$$

where *X*_*ik*_ is the *k*-th predictor, β_*k*_ is the coefficient of *X*_*ik*_, β_0_ is a constant, and ε_*i*_ is the error, which follows the logistic distribution.

### Local model: GWLR model

In stark contrast to traditional regression models that employ a single equation to describe the association between the predicted variable and predictors, the GWR model creates a battery of equations to account for the possible spatial heterogeneity in the relationship, making it possible to visualize broad patterns in the coefficient estimates. Furthermore, every point has a unique equation that is assessed using this point and its neighbors. Simply put, by relaxing the assumption of spatially invariant associations, the GWR model expands the classical regression framework and permits the estimate of point-varying parameters. It has been utilized in numerous empirical studies (53–57).

The GWLR model belongs to the GWR family and is represented as follows:


(5) $$\begin{array}{l} U_{i}=\beta_{0}\left(u_{i},\;v_{i}\right)+\sum_{k}\beta_{k}\left(u_{i},\;v_{i}\right)X_{ik}+\;\varepsilon_{i}, \end{array}$$

where (*u*_*i*_, *v*_*i*_) stands for the coordinates of point *i*, β_*k*_(*u*_*i*_, *v*_*i*_) denotes the coefficient of *X*_*ik*_, β_0_(*u*_*i*_, *v*_*i*_) is the constant of point *i*. β_*k*_(*u*_*i*_, *v*_*i*_) and β_0_(*u*_*i*_, *v*_*i*_) are parameters to be collaboratively calculated.

A kernel function is required to calculate the weights of nearby points for any given point. Four frequently employed functions are fixed Gaussian, adaptive Gaussian, fixed bi-square, and adaptive bi-square kernel functions. For the fixed Gaussian and fixed bi-square kernel functions, weights are allocated as a continuous function of distance. The adaptive Gaussian and adaptive bi-square kernel functions, in contrast, permit the geographical extent (bandwidth) to fluctuate over space rather than maintaining a constant bandwidth. The formulas of the four kernel functions are as follows.


(6) $$\begin{array}{l} \text{Fixed Gaussian kernel}:\;w_{ij}=\exp\left(-{d_{ij}}^{2}/\theta^{2}\right) \end{array}$$

(7) $$\begin{array}{l} \text{Adaptive Gaussian kernel}:\;w_{ij}=\exp\left(-{d_{ij}}^{2}/{\theta_{i\left(k\right)}}^{2}\right) \end{array}$$

(8) $$\text{Fixed bi}-\text{square kernel}:\;w_{ij}=\;\{\begin{array}{cc} {\left(1-d_{ij}{}^{2}/\theta^{2}\right)}^{2} & \text{if}d_{ij}<\theta \\ 0\; & \text{otherwise} \end{array}$$

(9) $$\begin{array}{l} \text{Adaptive bi-square kernel:}\\ w_{ij}=\{\begin{array}{cc} {\left(1-d_{ij}{}^{2}/\theta_{i(k)}{}^{2}\right)}^{2} & \text{if }d_{ij}<\theta_{i(k)}\\ 0\; & \text{otherwise} \end{array} \end{array}$$

where *w*_*ij*_ is point *j*'s weight for the local equation of point *i*, *d*_*ij*_ is the Euclidean distance between points *i* and *j*, θ is a fixed bandwidth, and θ_*i*(*k*)_ is an adaptive bandwidth depending on the *k*th nearest neighbor distance.

### Variables

**Figure 4:**
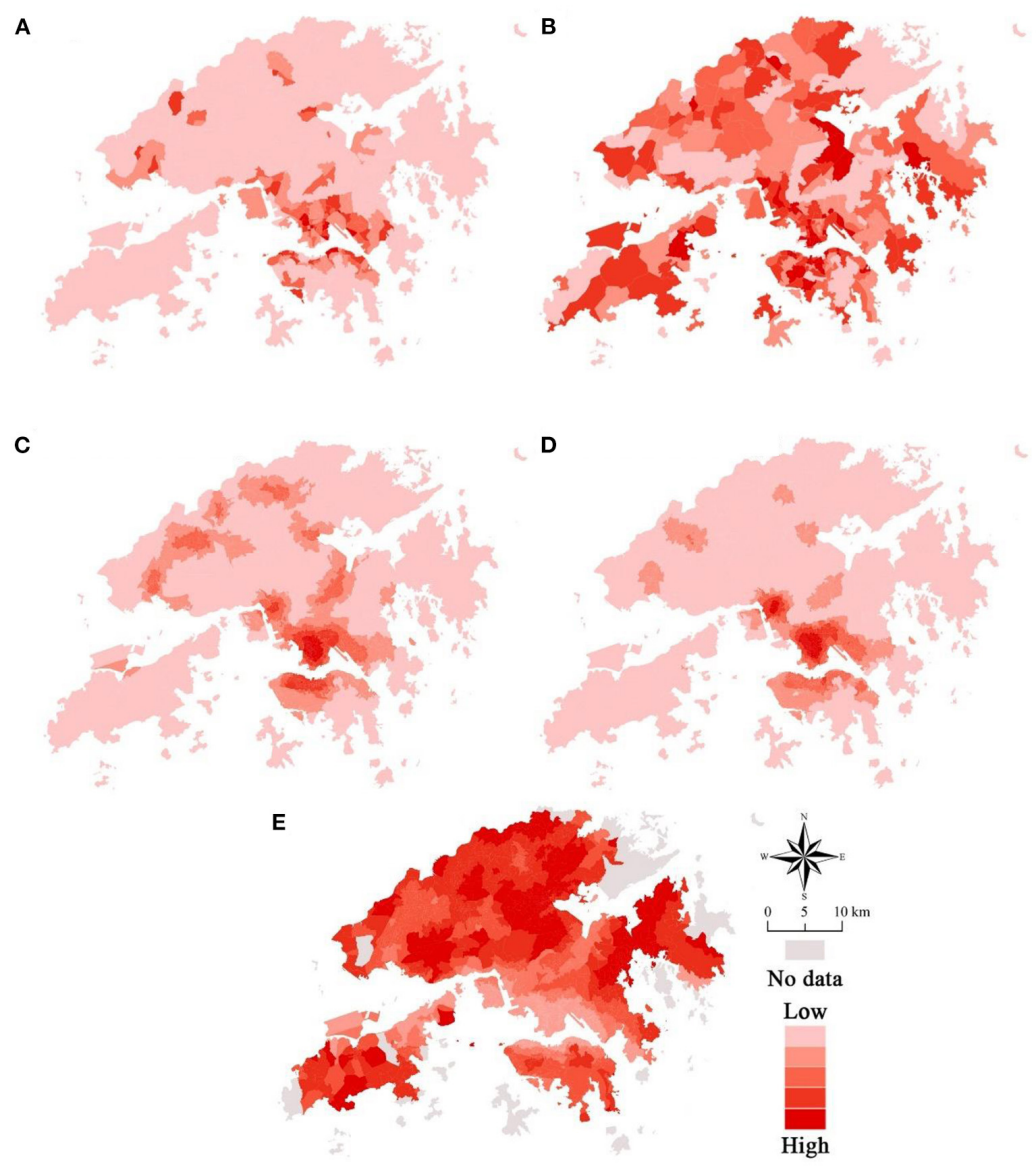
The spatial distribution of the five built environment attributes. **(A)** Population density. **(B)** Land-use mix. **(C)** Intersection density. **(D)** Access to bus stops. **(E)** Street greenery.

Eight predictors, which were comprised of three socio-demographic variables and five built environment variables, were selected in this study. The choice of built environment variables follows the “3Ds” built environment assessment framework (21). Table 1 shows the summary of the predictor and predicted variables. Figure 4 shows the spatial distribution of the five built environment attributes.

**Table 1:** Summary of the predicted variable and predictors.

**Variable**	**Description**	**Mean/proportion**	**Std. Dev**.
**Predicted variable**			
Propensity to walk	= 1 for having walked out in the reference 24 h, = 0 otherwise	0.63	
**Predictors: socio-demographics**			
Male	= 1 for male, = 0 for female	0.49	
Age	(unit: year)	73.82	6.93
Car	= 1 for a person with household car availability, = 0 otherwise	0.07	
**Predictors: built environment**			
Population density	Neighborhood-level population density (unit: 10^3^ people/km^2^)	47.98	32.95
Land-use mix	Entropy for neighborhood land uses.	0.44	0.23
Intersection density	Neighborhood-level street intersection density (unit: 1/km^2^)	72.14	49.75
Street greenery	Green view index	0.15	0.03
Access to bus stops	Number of bus stops in the neighborhood	20.10	11.34
Number of observations	10,700

## Results

**Figure 5:**
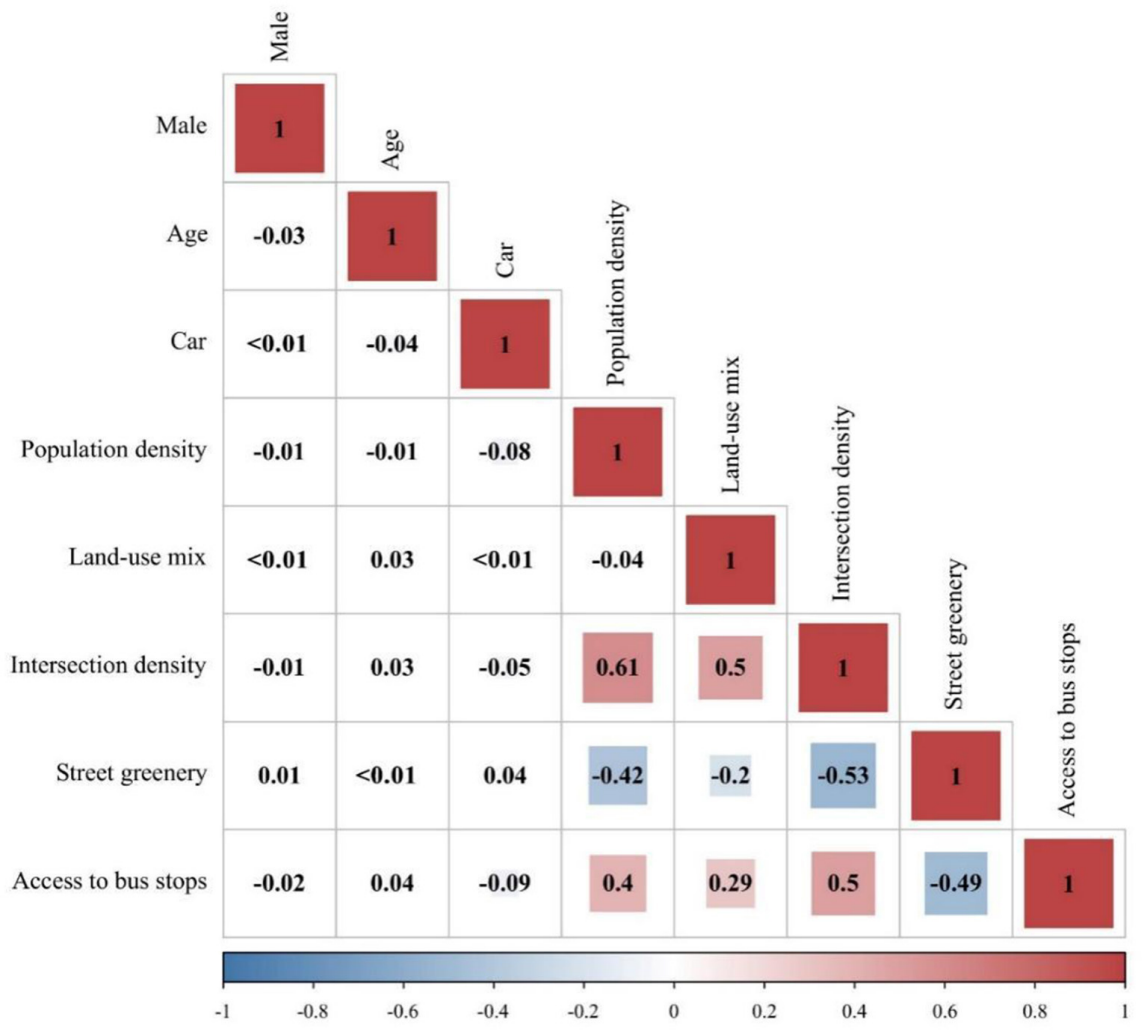
Correlation coefficients among the predictors.

A pair-wise correlation analysis was conducted to analyze whether collinearity exists among the predictors. Figure 5 shows the outcome. It reveals that Pearson's correlation coefficients are far smaller than 0.7, indicating the absence of collinearity.

Global and local modeling results are shown below.

### Global modeling results

A logistic regression model was developed herein to demonstrate the correlation between the built environment and the propensity to walk of older adults in Hong Kong. Table 2 displays the outcomes. Almost all predictors (control variables and explanatory variables) have a significant effect on this propensity. Specifically, population density is shown to increase the propensity to walk of older adults significantly. This outcome meets our expectations and reinforces earlier research conducted in the same city (42). A convincing explanation may be that high population density areas mean that pedestrians are allocated more road space and amenities, which makes walking easier, safer, and more enjoyable (58). Moreover, areas with high population density have various facilities (e.g., retail stores, chess rooms, and stores), which can better meet the travel needs (shopping, leisure, fitness, etc.) of older adults, thus promoting walking trips.

**Table 2:** Global modeling results.

**Variable**	**Coefficient**	**Std. Dev**.	***t*** **-statistic**	***p*** **-value**
Male	−0.285^**^	0.041	−6.938	0.000
Age	0.045^**^	0.003	14.347	0.000
Automobile	−0.400^**^	0.084	−4.781	0.000
household income	−0.020^**^	0.005	−4.215	0.000
Population density	0.056^**^	0.010	5.908	0.000
Land-use mix	0.314^*^	0.124	2.531	0.011
Intersection density	0.009	0.008	1.232	0.218
Street greenery	3.157^**^	0.748	4.223	0.000
Access to bus stops	0.009^**^	0.002	3.864	0.000
Constant	−3.595^**^	0.284	−12.648	0.000
**Performance statistic**
AIC	13,593.37
AICc	13,593.39

* Significant at the 5% level.

** Significant at the 1% level.

Land-use mix positively affects older adults' propensity to walk, reinforcing earlier research showing that older adults living in communities with a high land-use mix are more inclined to walk. This observation is because a high land-use mix typically indicates that destinations have abundant types of amenities that meet the walking requirements of older adults, hence encouraging walking trips.

Street greenery has a positive and statistically significant connection with older adults' walking trips at the 99% confidence interval. This outcome concurs with most of the evidence obtained from the existing literature (47, 59, 60), indicating that older residents living in neighborhoods with more greenery prefer to walk more.

A significant and positive correlation between bus stop accessibility and the propensity to walk of older adults is also identified. Older adults will typically walk to and from the bus stop if they need to travel by bus, thus increasing their walking trips (57). Furthermore, regions with high bus accessibility direct travelers to walk to transit stations, which create a walkable atmosphere and, from the perspective of perceived safety, potentially encourage people's walking trips (58).

However, intersection density has zero or unexpected correlation with aspects related to older adults' propensity to walk. This finding is significantly different from previous studies, particularly those conducted in East Asia (58). We should know that the above outcome merely presents an insignificant average effect of intersection density and thus may be, at least partially, attributable to spatial heterogeneity.

### Local modeling results

The abovementioned findings provide an answer on whether the built environment does affect older adults' propensity to walk. However, they cannot clarify whether there is spatial heterogeneity in this effect. Simply put, the global model can examine the average effect, but it cannot discern the potential spatial variation effect. Therefore, the GWLR model was employed in this study to uncover this effect. The software MGWR (version 2.2.1) (61) was applied to estimate the model.

Table 3 presents the outcomes of the regression model. The GWLR model has lower AIC and AICc than the logistic regression model. This observation indicates that the GWLR model outperforms the logistic regression model in fitting the data and justifies our shift from global models to local ones. Moreover, great variations in the coefficients of the built environment variables are also determined. Furthermore, the variation range for each predictor seems to be large. Positive and negative coefficients can be observed for each predictor, indicating that the variables exert positive effects in some regions and negative effects in others. This observation also means that the global regression estimation is inadequate and echoes the argument made by Mulley (62) that “inferring policy from a single, average, global value might well be misleading” (p. 1,722).

**Table 3:** Local modeling results.

**Variable**	**Coefficient**				
	**Mean**	**Std. Dev**.	**Min**	**Median**	**Max**
Male	−0.312	0.245	−1.218	−0.291	0.320
Age	0.050	0.025	−0.020	0.049	0.125
Automobile	−0.237	0.714	−3.337	−0.232	1.737
household income	−0.009	0.040	−0.104	−0.011	0.112
Population density	0.157	0.330	−2.650	0.077	1.259
Land-use mix	1.265	8.571	−28.259	1.186	119.403
Intersection density	−0.096	0.480	−4.079	−0.012	1.228
Street greenery	−8.866	49.562	−410.161	−7.134	109.353
Access to bus stops	0.014	0.032	−0.112	0.010	0.123
Constant	−2.702	7.141	−18.229	−3.375	43.185
**Performance statistic**			
AIC	13,113.53
AICc	13,137.73

**Figure 6:**
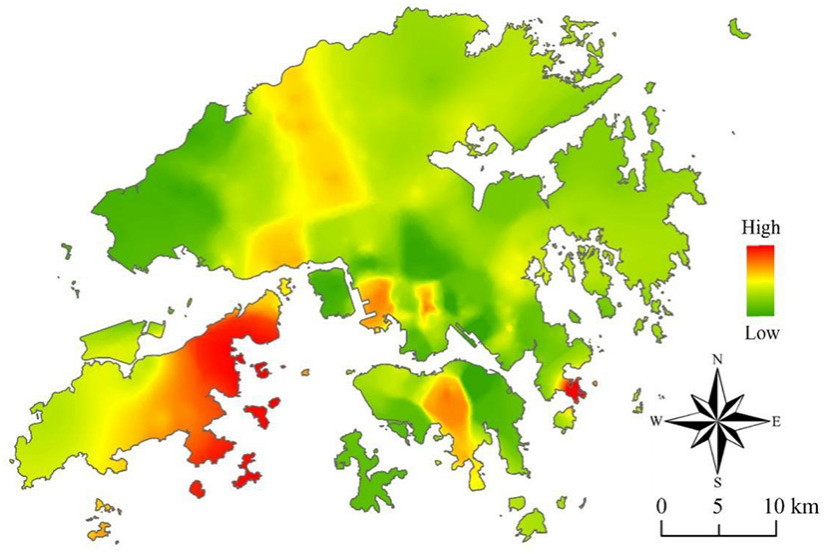
The spatial distribution of the proportion of the predicted variable variance that is accounted for by the predictors.

As noted above, GWR-family models, including the GWLR model, have the advantage of ease of visualization. Inverse distance weighted (IDW) interpolation is used to assign values to unknown points in the ArcGIS Pro platform. Figure 6 shows the spatial distribution of the percentage of deviance explained, namely the proportion of the predicted variable variance accounted for by the predictors. We observed that the deviance explained is not homogeneously distributed across space. For example, the deviance explained is highest in the east of Lantau Island, indicating that the relationships between the propensity to walk and the eight selected predictors can be better predicted in the east of Lantau Island, and that the propensity of older adults in other regions is more likely to be affected by other unobserved factors.

**Figure 7:**
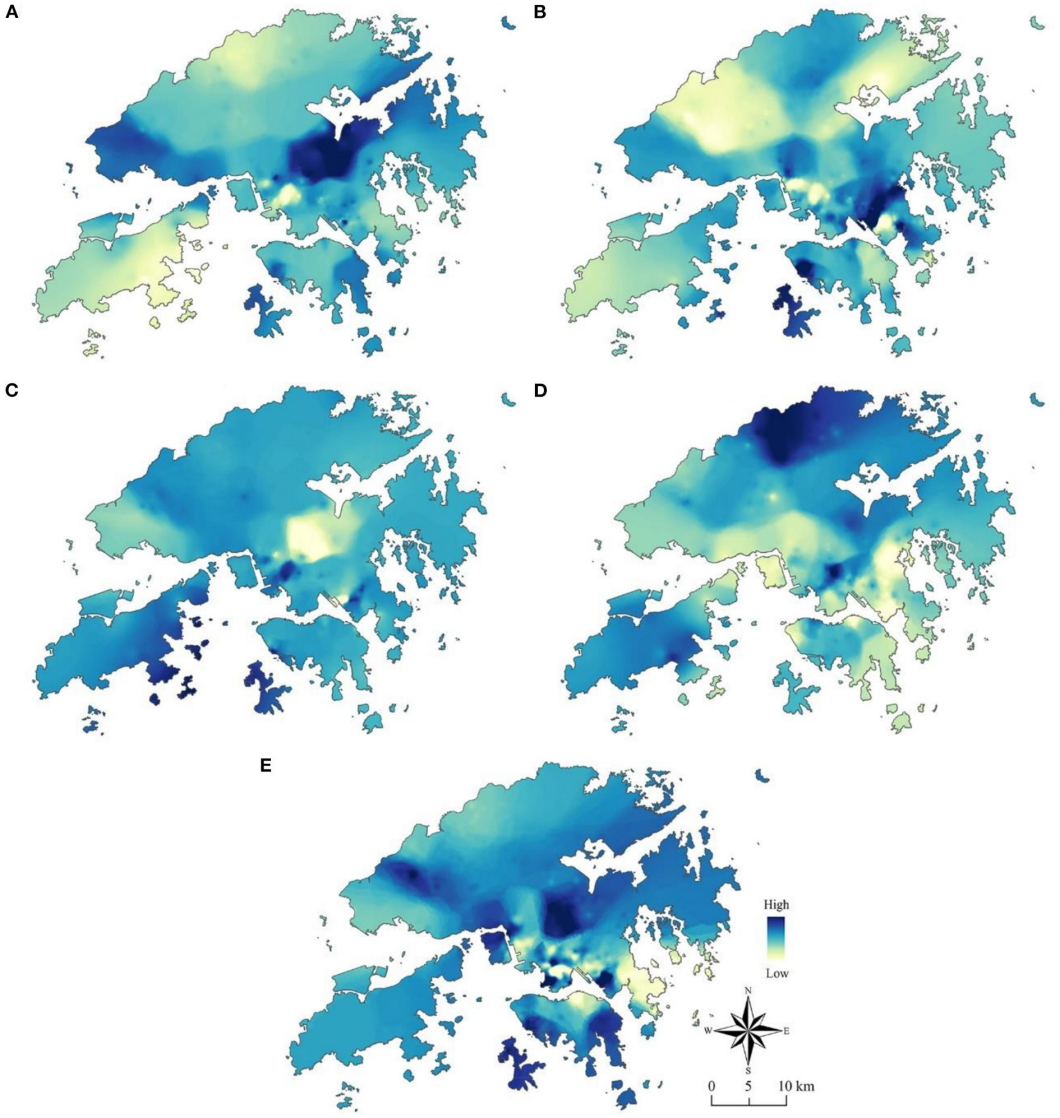
The spatial distribution of the coefficients of the built environment variables. **(A)** Coefficient of population density. **(B)** Coefficient of land-use mix. **(C)** Coefficient of intersection density. **(D)** Coefficient of access to bus stops. **(E)** Coefficient of street greenery.

The spatial distribution of the coefficients of the five built environment variables is of predominant interest herein. It is revealed in Figure 7, where IDW interpolation was once again adopted. We observed that the distribution patterns are highly irregular, but patterns for a variable remain inapplicable to other variables.

Figure 7 exhibits the effect of a predictor in affecting the predicted variable in each location. Population density has the largest effect in Shatin, followed by Tuen Mun. Access to bus stops has the greatest effect in Mong Kok and the areas adjacent to Lo Wu Port. Furthermore, intersection density has positive effects in places like the east of Lantau Island and Cheung Sha Wan but has negative effects in places such as Sha Tin and Tai Wai. This observation provides suggestive evidence for the insignificance of the average effect of intersection density.

## Conclusion and discussion

A rapidly aging global population will create varying degrees of socio-economic effects worldwide. Hong Kong, the region with the second-highest aging rate in Asia, undoubtedly faces this issue as well. The unstoppable rise in the number and share of the older population has drawn increasing concern from the government, society, and academia. Meanwhile, walking is among the most crucial travel modes for older adults, and insufficient walking activities have a clearly detrimental impact on their overall wellbeing. Therefore, it is important to consider the preferences and needs of older adults and turn the development of walkable built environments for older adults into viable areas of focus for future studies. This study looks at the spatially varying association between older adults' walking behavior (specifically, propensity to walk) and the built environment and aims to develop the latter to be able to satisfy older adults' walking demands and promote their walking trips. Based on HKTCS data and geo-data (e.g., GSV imagery), this study develops a logistic regression model and a GWLR model to determine how the built environment affects the propensity to walk of older adults and the effect's spatial heterogeneity. This study has found that older adults' propensity to walk was positively and significantly related to population density, land-use mix, street greenery, and access to bus stops, but it was insignificantly related to intersection density. Built environment attributes were also found to have spatially heterogeneous effects on walking propensity. Methodologically, this study assesses street greenery by applying big geographical data, demonstrating the great necessity of integrating big data into urban studies. Employing new/big data can greatly improve our understanding of the relationship between activity behavior and the built environment and is a valuable addition to traditionally built environment measurements, which should become an important perspective for future studies.

This study presents several theoretical, methodological, and practical implications for decision-makers, practitioners, and planners. First, the results can offer crucial theoretical backing for the development of senior-friendly built environments that promote their walking trips. Older adults' propensity to walk can be enhanced by increasing population density, land-use mix, and accessibility to bus stops. Moreover, the GSV-based measure of street greenery strongly influenced older adults' propensity to walk, indicating its direct ties to their daily walking trips. These four factors ought to be crucial in the planning of a senior-friendly built environment in the future to improve the living standards and wellbeing of older adults. Furthermore, the people-oriented urban design must consider older adults' preferences and behavior (63). As an age group with considerable leisure time, older adults (unlike younger people) rarely make mandatory trips (e.g., commuting) but often make optional ones. In China, chess rooms, parks, and grocery markets are essential destinations for older adults, and making them accessible by walking is recommended (37). Therefore, in the development of senior-friendly communities, policymakers should focus on attributes that are significantly relevant to promoting older adults' walking behavior. Finally, since most studies recently conducted concentrated primarily on the global (spatially fixed) associations between the built environment and older adults' travel behavior and ignored spatial heterogeneity, this study focusing on the local associations deepens our understanding of the spatially heterogeneous effect between the built environment and older adults' walking behavior.

Traditionally, due to the prevalence of regression-based correlation studies, policymakers and urban planners tend to assume unidirectional effects between built environment attributes and people's travel behavior (42). They prefer to singularly raise/lower a certain indicator and overlook the potential non-linear effects. For example, population density was once thought to affect walking behavior positively and monotonically. This is because high-density areas always imply well-equipped neighborhoods and facilities which encourage walking (42). However, many recent studies based on machine learning techniques have described the non-linear and threshold effects of the built environment, thus triggering a change in conventional wisdom. Cheng et al. (58) countered that population density above a particular threshold could have a detrimental impact on walking behavior because ultra-dense areas induce crowding and a higher risk of injury, thereby discouraging walking. Therefore, confining built environment variables to a reasonable (perhaps moderate) interval may be the most effective approach. Furthermore, nearly all machine learning techniques cannot model spatially varying relationships, although some exceptions exist (64). We believe the incorporation of spatial heterogeneity into the machine learning framework is an interesting future research direction.

A voluminous body of literature (including this study) has been devoted to teasing out the relationships between the “physical” dimension of the living/neighborhood environment (i.e., the natural and built environments) and activity behavior. Nevertheless, very limited scholarly attention has been paid to the “social” dimension of the environment (i.e., the social environment), which may also be a determinant of activity behavior. Currently, due to urban development patterns, changes in technology, and hectic lifestyles, people are always separated from their neighborhoods and communities and detached from their surroundings. Promoting social interaction, building a sense of community, and creating an inclusive and cohesive social environment have thus been extensively advocated. We suspect that many social environment variables, such as social cohesion and trust, social support, social interaction, and sense and inclusiveness of community, influence people's activity behavior. More studies are recommended to devote themselves to this issue, where profound policy and practical implications can be obtained. Summarily, we continue to urge that the definition of the neighborhood environment should be considerably broadened, arguing that the addition of the social dimension advances our knowledge of the relationships between the living environment and activity behavior.

Despite some highly insightful conclusions, this study inevitably has the following shortcomings. First, the models used in this study presupposed the global and local correlations between variables. However, employing machine learning models can accurately identify the complicated non-linear associations between older adults' walking behavior and the built environment without the need to pre-determine variable relationships (65). Second, this study identified correlations between variables but could not examine causality given the cross-sectional nature of HKTCS data. Therefore, conducting longitudinal surveys is needed to gather multi-wave first-hand data, obtain causality evidence, and develop more insightful conclusions. Third, this study exclusively concentrates on the role of the outdoor environment while ignoring the indoor environment. The indoor environment is important because people spend 80% of their time indoors (66, 67). It is likely to influence people's outdoor activity behavior. For example, if living in a cramped and congested home, a person may prefer to go outdoor. Fourth, older adults are arguably not a homogeneous group. For example, the oldest people may need rest facilities on the walking path, but younger seniors may not need them so urgently. Looking into senior subgroups (e.g., male vs. female) can determine diverse demands and preferences and obtain richer findings (68). Last, Hong Kong is well-known as an extremely compact, highly mixed-use metropolis. The extent to which the findings of this study are externally valid in mainland Chinese cities and beyond remains to be tested. Further empirical studies in various contexts are required to draw more thorough conclusions.

## Data availability statement

The original contributions presented in the study are included in the article/supplementary material, further inquiries can be directed to the corresponding author.

## Author contributions

CY: conceptualization, funding acquisition, supervision, and writing—original draft. XT: validation and writing—review and editing. LY: formal analysis, methodology, formal analysis, and writing—review and editing. All authors contributed to the article and approved the submitted version.

## Funding

This study was supported by the Sichuan Science and Technology Program (No. 2022JDR0178).

## Conflict of interest

The authors declare that the research was conducted in the absence of any commercial or financial relationships that could be construed as a potential conflict of interest.

## Publisher's note

All claims expressed in this article are solely those of the authors and do not necessarily represent those of their affiliated organizations, or those of the publisher, the editors and the reviewers. Any product that may be evaluated in this article, or claim that may be made by its manufacturer, is not guaranteed or endorsed by the publisher.

## References

[1] Zhang Y Cao M Cheng L Gao X De Vos J. Exploring the temporal variations in accessibility to health services for older adults: a case study in Greater London. J Transport Health. (2022) 24:101334. 10.1016/j.jth.2022.101334

[2] Department for Economic and Social Affairs of the United Nations. World Population Prospects 2019: Highlights. New York, NY. (2019).

[3] Yang Y Sasaki K Cheng L Tao S. Does the built environment matter for active travel among older adults: insights from Chiba City, Japan. J Transport Geogr. (2022) 101:103338. 10.1016/j.jtrangeo.2022.103338

[4] Van Den Berg P Kemperman A De Kleijn B Borgers A. Ageing and loneliness: the role of mobility and the built environment. Travel Behav Soc. (2016) 5:48–55. 10.1016/j.tbs.2015.03.001

[5] Frank LD Saelens BE Powell KE Chapman JE. Stepping towards causation: do built environments or neighborhood and travel preferences explain physical activity, driving, and obesity? Soc Sci Med. (2007) 65:1898–914. 10.1016/j.socscimed.2007.05.053 17644231

[6] Sælensminde K. Cost–benefit analyses of walking and cycling track networks taking into account insecurity, health effects and external costs of motorized traffic. Transport Res A. (2004) 38:593–606. 10.1016/j.tra.2004.04.003

[7] Michel J-P Leonardi M Martin M Prina M. WHO's report for the decade of healthy ageing 2021–30 sets the stage for globally comparable data on healthy ageing. Lancet Healthy Longevity. (2021) 2:e121–e2. 10.1016/S2666-7568(21)00002-7 36098109

[8] Rudnicka E Napierała P Podfigurna A Meczekalski B Smolarczyk R Grymowicz M. The World Health Organization (WHO) approach to healthy ageing. Maturitas. (2020) 139:6–11. 10.1016/j.maturitas.2020.05.018 32747042 PMC7250103

[9] Liu Z Kemperman A Timmermans H. Influence of neighborhood characteristics on physical activity, health, and quality of life of older adults: a path analysis. Front Public Health. (2021) 9:783510. 10.3389/fpubh.2021.783510 34900922 PMC8652252

[10] Li J Tian L Ouyang W. Exploring the relationship between neighborhood-built environment and elderly health: a research based on heterogeneity of age and gender groups in Beijing. Front Public Health. (2022) 10:882361. 10.3389/fpubh.2022.882361 35712265 PMC9194851

[11] Cheng L Chen X Yang S Cao Z De Vos J Witlox F. Active travel for active ageing in China: the role of built environment. J Transport Geogr. (2019) 76:142–52. 10.1016/j.jtrangeo.2019.03.01034113188

[12] Liu Z Kemperman A Timmermans H Yang D. Heterogeneity in physical activity participation of older adults: a latent class analysis. J Transport Geogr. (2021) 92:102999. 10.1016/j.jtrangeo.2021.10299928143458

[13] Althoff T Sosič R Hicks JL King AC Delp SL Leskovec J. Large-scale physical activity data reveal worldwide activity inequality. Nature. (2017) 547:336–9. 10.1038/nature23018 28693034 PMC5774986

[14] Tu M Li W Orfila O Li Y Gruyer D. Exploring nonlinear effects of the built environment on ridesplitting: evidence from Chengdu. Transport Res D. (2021) 93:102776. 10.1016/j.trd.2021.102776

[15] Chen S Bao Z Lou V. Assessing the impact of the built environment on healthy aging: a gender-oriented Hong Kong study. Environ Impact Assess Rev. (2022) 95:106812. 10.1016/j.eiar.2022.106812

[16] Li W Pu Z Li Y Tu M. How does ride splitting reduce emissions from ride sourcing? A spatiotemporal analysis in Chengdu, China. Transport Res D. (2021) 95:102885. 10.1016/j.trd.2021.102885

[17] Chen S Wang T Bao Z Lou V, A. path analysis of the effect of neighborhood built environment on public health of older adults: a Hong Kong study. Front Public Health. (2022) 10:861836. 10.3389/fpubh.2022.861836 35359794 PMC8964032

[18] Ao Y Yang D Chen C Wang Y. Effects of rural built environment on travel-related CO_2_ emissions considering travel attitudes. Transport Res D. (2019) 73:187–204. 10.1016/j.trd.2019.07.004

[19] Ao Y Yang D Chen C Wang Y. Exploring the effects of the rural built environment on household car ownership after controlling for preference and attitude: evidence from Sichuan, China. J Transport Geogr. (2019) 74:24–36. 10.1016/j.jtrangeo.2018.11.002

[20] Yang L Tang X Yang H Meng F Liu J. Using a system of equations to assess the determinants of the walking behavior of older adults. Trans. GIS (2022) 26:1339–54. 10.1111/tgis.12916

[21] Cervero R Kockelman K. Travel demand and the 3Ds: density, diversity, and design. Transport Res D. (1997) 2:199–219. 10.1016/S1361-9209(97)00009-6

[22] Ewing R Cervero R. Travel and the built environment: a meta-analysis. J Am Plan Assoc. (2010) 76:265–94. 10.1080/01944361003766766

[23] Day K. Built environmental correlates of physical activity in China: a review. Prev Med Rep. (2016) 3:303–16. 10.1016/j.pmedr.2016.03.007 27419030 PMC4929152

[24] Wu J Zhao C Li C Wang T Wang L Zhang Y. Non-linear relationships between the built environment and walking frequency among older adults in Zhongshan, China. Front Public Health. (2021) 9:686144. 10.3389/fpubh.2021.686144 34422746 PMC8374739

[25] Troped PJ Starnes HA Puett RC Tamura K Cromley EK James P. Relationships between the built environment and walking and weight status among older women in three US States. J Aging Phys Act. (2014) 22:114–25. 10.1123/japa.2012-0137 23538637 PMC4186705

[26] Roe J Mondschein A Neale C Barnes L Boukhechba M Lopez S. The urban built environment, walking and mental health outcomes among older adults: a pilot study. Front Public Health. (2020) 8:575946. 10.3389/fpubh.2020.575946 33072714 PMC7538636

[27] Kaczynski AT Potwarka LR Smale BJ Havitz ME. Association of parkland proximity with neighborhood and park-based physical activity: variations by gender and age. Leisure Sci. (2009) 31:174–91. 10.1080/01490400802686045

[28] Yang H Zhang Y Zhong L Zhang X Ling Z. Exploring spatial variation of bike sharing trip production and attraction: a study based on Chicago's Divvy system. Appl Geogr. (2020) 115:102130. 10.1016/j.apgeog.2019.102130

[29] Yang L Liu J Liang Y Lu Y Yang H. Spatially varying effects of street greenery on walking time of older adults. Int J Geo-Information. (2021) 10:596. 10.3390/ijgi10090596

[30] Yang L Liu J Lu Y Ao Y Guo Y Huang W. Global and local associations between urban greenery and travel propensity of older adults in Hong Kong. Sustain Cities Soc. (2020) 63:102442. 10.1016/j.scs.2020.102442

[31] Yang L Cui X. Determinants of elderly mobility in Hong Kong: implications for elderly-friendly transport. China City Plan Rev. (2020) 29:74–83.

[32] Kim S. Analysis of elderly mobility by structural equation modeling. Transport Res Record. (2003) 1854:81–9. 10.3141/1854-09

[33] Paez A Scott D Potoglou D Kanaroglou P Newbold KB. Elderly mobility: demographic and spatial analysis of trip making in the Hamilton CMA, Canada. Urban Stud. (2007) 44:123–46. 10.1080/00420980601023885

[34] Schmöcker J-D Quddus MA Noland RB Bell MG. Estimating trip generation of elderly and disabled people: analysis of London data. Transport Res Record. (2005) 1924:9–18. 10.1177/0361198105192400102

[35] Kim S Ulfarsson GF. Travel mode choice of the elderly: effects of personal, household, neighborhood, and trip characteristics. Transport Res Record. (2004) 1894:117–26. 10.3141/1894-13

[36] Evans EL. Influences on mobility among non-driving older Americans. Transport Res Circular E-C026. (2001) 2001:151–68.

[37] Feng J. The influence of built environment on travel behavior of the elderly in urban China. Transport Res D. (2017) 52:619–33. 10.1016/j.trd.2016.11.003

[38] Feng J Dijst M Wissink B Prillwitz J. The impacts of household structure on the travel behaviour of seniors and young parents in China. J Transport Geogr. (2013) 30:117–26. 10.1016/j.jtrangeo.2013.03.008

[39] Feng J Dijst M Wissink B Prillwitz J. Elderly co-residence and the household responsibilities hypothesis: evidence from Nanjing, China. Urban Geogr. (2015) 36:757–76. 10.1080/02723638.2015.1039407

[40] Schwanen T Dijst M Dieleman FM. Leisure trips of senior citizens: determinants of modal choice. Tijdschrift voor economische en sociale geografie. (2001) 92:347–60. 10.1111/1467-9663.00161

[41] Roorda MJ Páez A Morency C Mercado R Farber S. Trip generation of vulnerable populations in three Canadian cities: a spatial ordered probit approach. Transportation. (2010) 37:525–48. 10.1007/s11116-010-9263-3

[42] Yang L Ao Y Ke J Lu Y Liang Y. To walk or not to walk? Examining non-linear effects of streetscape greenery on walking propensity of older adults. J Transport Geogr. (2021) 94:103099. 10.1016/j.jtrangeo.2021.103099

[43] Truong LT Somenahalli SV. Exploring frequency of public transport use among older adults: a study in Adelaide, Australia. Travel Behav Soc. (2015) 2:148–55. 10.1016/j.tbs.2014.12.004

[44] Mercado R Páez A. Determinants of distance traveled with a focus on the elderly: a multilevel analysis in the Hamilton CMA, Canada. J Transport Geogr. (2009) 17:65–76. 10.1016/j.jtrangeo.2008.04.012

[45] Su F Bell MG. Transport for older people: characteristics and solutions. Res Transport Econ. (2009) 25:46–55. 10.1016/j.retrec.2009.08.006

[46] Pettersson P Schmöcker J-D. Active ageing in developing countries?–trip generation and tour complexity of older people in Metro Manila. J Transport Geogr. (2010) 18:613–23. 10.1016/j.jtrangeo.2010.03.015

[47] Yang Y He D Gou Z Wang R Liu Y Lu Y. Association between street greenery and walking behavior in older adults in Hong Kong. Sustain Cities Soc. (2019) 51:101747. 10.1016/j.scs.2019.10174729758477

[48] Cheng L De Vos J Shi K Yang M Chen X Witlox F. Do residential location effects on travel behavior differ between the elderly and younger adults? Transport Res D. (2019) 73:367–80. 10.1016/j.trd.2019.07.015

[49] Kang Y Zhang F Gao S Lin H Liu Y. A review of urban physical environment sensing using street view imagery in public health studies. Ann GIS. (2020) 26:261–75. 10.1080/19475683.2020.1791954

[50] Biljecki F Ito K. Street view imagery in urban analytics and GIS: a review. Landsc Urban Plan. (2021) 215:104217. 10.1016/j.landurbplan.2021.104217

[51] Bai Y Cao M Wang R Liu Y Wang S. How street greenery facilitates active travel for university students. J Transport Health. (2022) 26:101393. 10.1016/j.jth.2022.101393

[52] Long J Shelhamer E Darrell T. Fully convolutional networks for semantic segmentation. In: Proceedings of the IEEE Conference on Computer Vision and Pattern Recognition, Boston, MA, USA, (2015). 10.1109/CVPR.2015.7298965 27244717

[53] Tang J Gao F Han C Cen X Li Z. Uncovering the spatially heterogeneous effects of shared mobility on public transit and taxi. J Transport Geogr. (2021) 95:103134. 10.1016/j.jtrangeo.2021.103134

[54] Li W Chen S Dong J Wu J. Exploring the spatial variations of transfer distances between dockless bike-sharing systems and metros. J Transport Geogr. (2021) 92:103032. 10.1016/j.jtrangeo.2021.103032

[55] Xu P Huang H. Modeling crash spatial heterogeneity: random parameter versus geographically weighting. Accid Anal Prev. (2015) 75:16–25. 10.1016/j.aap.2014.10.020 25460087

[56] Yang J Bao Y Zhang Y Li X Ge Q. Impact of accessibility on housing prices in Dalian city of China based on a geographically weighted regression model. Chinese Geograph Sci. (2018) 28:505–15. 10.1007/s11769-018-0954-6

[57] Liu Q Zhao P Xiao Y Zhou X Yang J. Walking accessibility to the bus stop: does it affect residential rents? The case of Jinan, China. Land. (2022) 11:860. 10.3390/land11060860

[58] Cheng L De Vos J Zhao P Yang M Witlox F. Examining non-linear built environment effects on elderly's walking: a random forest approach. Transport Res D. (2020) 88:102552. 10.1016/j.trd.2020.102552

[59] Barnett DW Barnett A Nathan A Van Cauwenberg J Cerin E. Built environmental correlates of older adults' total physical activity and walking: a systematic review and meta-analysis. Int J Behav Nutr Phys Act. (2017) 14:1–24. 10.1186/s12966-017-0558-z 28784183 PMC5547528

[60] Zang P Liu X Zhao Y Guo H Lu Y Xue CQ. Eye-level street greenery and walking behaviors of older adults. Int J Environ Res Public Health. (2020) 17:6130. 10.3390/ijerph17176130 32846869 PMC7503975

[61] Oshan TM Li Z Kang W Wolf LJ Fotheringham AS. mgwr: a Python implementation of multiscale geographically weighted regression for investigating process spatial heterogeneity and scale. Int J Geo-Information. (2019) 8:269. 10.3390/ijgi8060269

[62] Mulley C. Accessibility and residential land value uplift: identifying spatial variations in the accessibility impacts of a bus transitway. Urban Stud. (2014) 51:1707–24. 10.1177/0042098013499082

[63] Jing C Bryan-Kinns N Yang S Zhi J Zhang J. The influence of mobile phone location and screen orientation on driving safety and the usability of car-sharing software in-car use. Int J Ind Ergon. (2021) 84:103168. 10.1016/j.ergon.2021.103168

[64] Zhang X Zhao X. Machine learning approach for spatial modeling of ridesourcing demand. J Transport Geogr. (2022) 100:103310. 10.1016/j.jtrangeo.2022.103310

[65] Lu W Chen J. Computer vision for solid waste sorting: a critical review of academic research. Waste Manag. (2022) 142:29–43. 10.1016/j.wasman.2022.02.009 35172271

[66] Gu Z Su S Lu W Yao Y. Estimating spatiotemporal contacts between individuals in underground shopping streets based on multi-agent simulation. Front Phys. (2022) 10:882904. 10.3389/fphy.2022.882904

[67] Gu Z Osaragi T Lu W. Simulating pedestrians' spatio-temporal distribution in underground spaces. Sustain Cities Soc. (2019) 48:101552. 10.1016/j.scs.2019.101552

[68] Yang Y Sasaki K Cheng L Liu X. Gender differences in active travel among older adults: non-linear built environment insights. Transport Res D. (2022) 110:103405. 10.1016/j.trd.2022.103405

